# Phenotypic Characterization and Comparative Genomics of the Melanin-Producing Yeast *Exophiala lecanii-corni* Reveals a Distinct Stress Tolerance Profile and Reduced Ribosomal Genetic Content

**DOI:** 10.3390/jof7121078

**Published:** 2021-12-15

**Authors:** Jillian Romsdahl, Zachary Schultzhaus, Christina A. Cuomo, Hong Dong, Hashanthi Abeyratne-Perera, W. Judson Hervey, Zheng Wang

**Affiliations:** 1 National Research Council Postdoctoral Research Associate, U.S. Naval Research Laboratory, Washington, DC 20375, USA; jillianromsdahl@gmail.com; 2 Center for Biomolecular Sciences and Engineering, U.S. Naval Research Laboratory, Washington, DC 20375, USA; zach.schultzhaus@gmail.com (Z.S.); judson.hervey@nrl.navy.mil (W.J.H.IV); 3 Infectious Disease and Microbiome Program, Broad Institute of MIT and Harvard, Cambridge, MA 02142, USA; cuomo@broadinstitute.org; 4 Biotechnology Branch, CCDC Army Research Laboratory, Adelphi, MD 20783, USA; hong.dong.civ@army.mil; 5 American Society for Engineering Education Postdoctoral Research Associate, U.S. Naval Research Laboratory, Washington, DC 20375, USA; hashanthi.ann@gmail.com

**Keywords:** *Exophiala lecanii-corni*, black yeast, melanin biosynthesis, extremophile, toluene degradation, comparative genomics

## Abstract

The black yeast *Exophiala lecanii-corni* of the order Chaetothyriales is notable for its ability to produce abundant quantities of DHN-melanin. While many other *Exophiala* species are frequent causal agents of human infection, *E. lecanii-corni* CBS 102400 lacks the thermotolerance requirements that enable pathogenicity, making it appealing for use in targeted functional studies and biotechnological applications. Here, we report the stress tolerance characteristics of *E. lecanii-corni*, with an emphasis on the influence of melanin on its resistance to various forms of stress. We find that *E. lecanii-corni* has a distinct stress tolerance profile that includes variation in resistance to temperature, osmotic, and oxidative stress relative to the extremophilic and pathogenic black yeast *Exophiala dermatitidis*. Notably, the presence of melanin substantially impacts stress resistance in *E. lecanii-corni*, while this was not found to be the case in *E. dermatitidis*. The cellular context, therefore, influences the role of melanin in stress protection. In addition, we present a detailed analysis of the *E. lecanii-corni* genome, revealing key differences in functional genetic content relative to other ascomycetous species, including a significant decrease in abundance of genes encoding ribosomal proteins. In all, this study provides insight into how genetics and physiology may underlie stress tolerance and enhances understanding of the genetic diversity of black yeasts.

## 1. Introduction

Black yeasts belonging to the ascomycete order Chaetothyriales have been of historic interest due to their frequent characterization as opportunistic pathogens [1]. These organisms, including those within the *Exophiala* genus, are distinguished by their abundant melanin content, thick cell walls, polymorphic growth, and ability to withstand a range of extreme conditions, including high and low temperatures, high salinity, desiccation, and various forms of non-ionizing and ionizing radiation [2,3,4,5]. Despite this, closely related species of black yeasts can also differ significantly with regard to pathogenicity and other attributes [1,6]. For example, *Exophiala lecanii-corni* is unable to grow at human physiological temperatures, and therefore is less pathogenic than *Exophiala dermatitidis*, being isolated from clinical settings approximately four times less frequently [7]. In addition, *E. lecanii-corni* CBS 102400, which was originally isolated from a bioreactor that aimed to decontaminate a toluene-containing waste gas stream [8], has been characterized as Biosafety Level 1 (BSL1), indicating that it is safe to work with and increasing its feasibility for use in biomanufacturing or other biotechnological applications. In recent decades, *E. lecanii-corni* has also garnered attention due to its demonstrated capacity to efficiently eliminate volatile organic compounds in vapor-phase, indicating its potential for bioremediation applications [8,9]. Importantly, the genetic tractability of this organism has already been demonstrated [10], which paves the way for targeted functional studies, as well as genetic optimization of production yields for bioproducts of interest.

*E. lecanii-corni* is notable for its constitutive production of large quantities of melanin via the 1,8-dihydroxynaphthalene (DHN)-melanin pathway, which are embedded in the cell wall and also released in culture filtrate [10]. Melanin has been reported to play a variety of roles in fungal systems, including the conferral of tolerance to various forms of stress, such as temperature, desiccation, and osmotic shock [11,12,13]. In some fungal species, melanin is also required for normal cell development, including accurate assembly of the cell wall and differentiation into secondary hyphae and conidia [14,15]. In addition, melanin contributes to the virulence of fungi by enhancing their ability to elude host immune responses [16,17]. The functional versatility of melanin within biological systems is a reflection of the broad variability of its physico-chemical properties, which are largely dictated by melanin synthesis and polymerization conditions that can lead to differences in oligomer size, molecular scaffold diversity, redox state, and supramolecular structure, resulting in different melanin variants with distinct characteristics [18]. These properties include antioxidant and free radical scavenging [19,20], photoprotection [21], semiconductivity [22], and metal and drug binding [23,24], which has led to the proposed use of melanin in bioelectronics, medical applications, materials science, and environmental remediation [25,26].

Phenotypic characterization coupled with comparative genomic analysis can strengthen our understanding of the genetic underpinnings that define variation among black yeasts. Our research group has previously conducted numerous studies into the black yeast *E. dermatitidis* that have advanced our understanding of the molecular basis of its extremophilic characteristics, as well as the impact of melanin on such processes [6,27,28,29,30]. In a previous study, we observed that although *E. dermatitidis* produces substantially less melanin than *E. lecanii-corni*, *E. dermatitidis* is far more resistant to high-dose acute ionizing radiation [6]. Here, we expand on this finding to evaluate differences in stress tolerance between these two species, as well as the impact of melanin on those processes. We find that there are substantive differences in resistance to temperature, osmotic stress, and oxidative stress between *E. lecanii-corni* and *E. dermatitidis*, suggesting that even among these closely related species of black yeast, the influence of melanin on stress resistance is not uniform. In addition, we report a comparative genomic analysis of *E. lecanii-corni* in relation to other ascomycetous species, revealing key differences in functional content, including significant underrepresentation of genes encoding ribosomal proteins. Together, this investigation represents the first detailed phenotypic and genomic characterization of *E. lecanii-corni* and contributes to the growing body of knowledge that continues to illuminate the genetic diversity of black yeast.

## 2. Materials and Methods

### 2.1. Cultivation of Fungi

The *E. lecanii-corni* strains used in this study were derived from the wild-type strain CBS 102400, and the *E. dermatitidis* strains used in this study were obtained from a previous study and derived from standard laboratory strain 8656 [6]. Unless otherwise noted, all strains were cultured at 25 °C in liquid or solid yeast peptone dextrose (YPD; 10 g/L yeast extract, 20 g/L peptone, 20 g/L glucose), with liquid cultures shaking at 225 RPM.

To identify culture media conditions that optimize melanin production yields, *E. lecanii-corni* was cultured using a range of standard fungal media recipes, including liquid YPD, yeast extract glucose (YEG; 5 g/L yeast extract, 20 g/L glucose, 1 mL/L Hutner’s trace elements), yeast extract sucrose (YES; 20 g/L yeast extract, 100 g/L sucrose, 1 mL/L Hutner’s trace elements), malt peptone glucose (MPG; 20 g/L malt extract, 1 g/L peptone, 20 g/L glucose), malt broth (MB; 130 g/L malt extract), Czapek (CZ; 30 g/L sucrose, 3 g/L NaNO_3_, 1 g/L K_2_HPO_4_, 0.5 g/L MgSO_4_, 0.5 g/L KCl, 0.01 g FeSO_4_), Czapek Yeast (CY; 5 g/L yeast extract, 30 g/L sucrose, 3 g/L NaNO_3_, 1 g/L K_2_HPO_4_, 0.5 g/L MgSO_4_, 0.5 g/L KCl, 0.01 g FeSO_4_), glucose minimal media optimized for yeast (GMM; 5.4 g/L glucose, 4 g/L KH_2_PO_4_, 1.20 g/L MgSO_4_, 1 g/L glycine, 0.8 mg/L thiamine, pH 5.5).

To screen cultures for melanin production, freshly cultured *E. lecanii-corni* cells were collected and inoculated at 10^4^ cells/mL into 250 mL flasks that each contained 100 mL of culture media to be screened. *E. lecanii-corni* cells were cultured at 25 °C while shaking at 200 RPM for 2 weeks, at which point the cells should be in stationary phase.

### 2.2. Isolation of Fungal Melanin

Fungal melanin was isolated from cultures screened in various growth conditions following 2 weeks of growth. To extract crude melanin, cells were spun down (10,000× *g*, 20 min) and the supernatant was transferred to a new Falcon tube. The supernatant was then acidified to a pH of 3 using 0.6 M HCl and kept at room temperature for 24 h, and then autoclaved to fully precipitate the remaining melanin. The precipitated crude melanin was then collected by centrifugation at (10,000× *g*, 10 min), washed with distilled water, and centrifuged again. This step was repeated until a neutral pH was achieved. Crude melanin was dried using a Savant SpeedVac Concentrator and the dry weight was measured. Isolation and purification of fungal melanin ghosts follows the method described by Dadachova [31].

Melanin ghosts were stained with 1% phosphotungstic acid aqueous solution (Electron Microscopy Sciences) for 2 h, spun down and freeze-dried, and were then imaged using a FEI Quanta 200 F field emission scanning electron microscope (FESEM) at an accelerating voltage of 5 kV.

Melanin ghosts were fixed with 4% paraformaldehyde for 2 h, stained with 1% osmium tetroxide for 1 h, centrifuged, and the pellets were freeze-dried. The dried samples were embedded in epoxy resin (low viscosity embedding kit) (Electron Microscopy Sciences, Hatfield, PA, USA) and cured overnight at 60 °C in an oven. Samples for TEM were prepared using a Leica EM UC7 ultramicrotome with a diamond knife. Sections were cut from the embedded samples at room temperature to a thickness of approximately 100 nm and collected on the TEM copper grids. The sections were imaged with a JEOL JEM-ARM200F transmission electron microscope operated at 200 kV.

### 2.3. Genetic Manipulation

The *E. lecanii-corni Elpks1Δ* mutant strain deficient in melanin production was generated using established gene deletion techniques [10,32]. Gene deletion cassettes were created by amplifying approximately 1 kb of the upstream and downstream genetic sequence flanking the target gene, which were fused to each end of the hygromycin resistance maker using the double-joint PCR technique [32]. The resulting construct was then transformed into *E. lecanii-corni* CBS 102400 using a previously described protocol [10]. In brief, cells were cultured in 300 mL YPD at a concentration of 10^5^ cells/mL for 36 h. Cells were then harvested and hyphae was removed via filtration with a sterile Miracloth. The resulting filtrate was chilled on ice for at least 30 min and cells were collected via centrifugation (4000× *g*, 8 min). The cell pellet was washed with cold water twice and resuspended in cold water at a concentration of 10^9^ cells/mL. Approximately 100 μL of the competent cells were combined with the DNA construct (3–4 μg DNA in a volume of 10 μL or less), mixed thoroughly, transferred to a chilled 0.2 cm electroporation cuvette, and subjected to electroporation using a Bio-Rad MicroPulser with default settings for fungi. The electroporated cells were transferred to 1 mL of liquid YPD, where they were incubated at 25 °C while shaking at 225 RPM for 3 h. The cells were then spread onto YPD plates containing 100 μg/mL hygromycin, which were grown at 25 °C until transformants were visible. Correct transformants were identified by observing a loss of color (due to lack of melanin production), which then underwent further confirmation via PCR (data not shown). The *E. dermatitidis pks1Δ* deletion strain was obtained from a previous study [6].

### 2.4. Cell Survival and Sensitivity Assays

For radiation resistance and stress sensitivity assays, cells were cultured in 2.5 mL YPD in 15 mL culture tubes for 6 days. Following growth, cells were re-suspended to a concentration of 10^8^ cells/mL in cold water and placed on ice. For UV-C exposure, cells were plated on YPD solid media and exposed in triplicate to either 0, 25, 50, or 75 mJ/cm^2^ of UV-C light within a biosafety cabinet with a dose rate of 0.273 mJ cm^−2^ s^−1^. For γ-radiation exposure, 100 μL of cells were aliquoted to PCR tubes, which were exposed to either 0, 500, 1000, or 1500 Gy of γ-radiation by a Cobalt-60 source with a dose rate of approximately 36 Gy/min. For each sample and dose, two biological replicates were exposed to the radiation source and each biological replicate was plated onto two YPD plates following the irradiation. For both UV-C and γ-radiation survival assays, relative survival was evaluated by counting colonies and determining CFU measurements. The standard deviations were generated with Microsoft Excel. For stress sensitivity, 2 μL droplets of cells were spotted in serial 10^0^–10^5^-fold dilutions on YPD plates with the appropriate concentration of stress-inducing agent (0.5 M NaCl or 7.5 mM H_2_O_2_). Unless otherwise noted, all culture were grown at 25 °C. Desiccation resistance was evaluated by transferring 100 μL of washed cells at a concentration 10^8^ cells/mL into a well of a sterile 96-well culture plate. The plate was placed in a sealed plastic container that also contained calcium sulfate desiccant and incubated at 30 °C for 1 week. The cells were resuspended in 100 μL of sterile water, diluted 10^0^–10^5^, and spotted in serial dilutions on YPD plates. Each stress assay was performed at least two times with two different biological replicates.

### 2.5. Phylogenetic and Comparative Genomic Analysis

The genome sequence and assembly of *E. lecanii-corni* was recently reported by our research group in collaboration with the Broad Institute [33]. Genes were predicted using BRAKER, which executed Genemark-ET with the parameter “fungus” [34,35]. tRNAs were predicted using tRNAscan [36] and rRNAs were predicted using RNAmmer [37]. Genes harboring Pfam domains that are present in repetitive elements or overlapping tRNA/rRNA features were removed. Genes were named and numbered sequentially. For comparative analyses, protein FASTA sequences for *E. dermatitidis* [27], *Exophiala mesophila* [38], *Exophiala sideris*, *Exophiala xenobiotica*, *Exophiala aquamarina*, *Exophiala oligosperma*, *Exophiala spinifera*, *Fonsecaea pedrosoi*, *Cladophialophora bantiana*, *Cladophialophora immunda* [5], *Aspergillus fumigatus* [39], *Aspergillus nidulans* [40], *Aspergillus niger* [41], *Trichophyton rubrum* [42], *Coccidioides immitis* [43], *Saccharomyces cerevisiae* [44], and *Schizosaccharomyces pombe* [45] were obtained from the National Center for Biotechnology Information (NCBI) web portal. Using these protein sequences, a rooted species tree was inferred using OrthoFinder [46,47], which involved prediction of orthogroups using the OrthoFinder algorithm, followed by inference of unrooted gene trees for each orthogroup using DendroBLAST [48], inference of an unrooted species tree using the STAG algorithm [49], and inference of the rooted species tree using the STRIDE algorithm [50].

Repeat elements were identified using RepeatModeler version 2.0.1 [51] and RepeatMasker version 4.1.0 [52], followed by classification using a 2017 RepBase RepeatMasker repetitive DNA element library [53]. For functional analysis, Pfam database protein families [54] were identified in all 18 species using the PfamScan Perl script (ftp://ftp.sanger.ac.uk/pub/databases/Pfam/Tools/ accessed on 11 November 2020). OrthoVenn2 was used to identify and annotate orthologous gene clusters and to generate Venn diagrams that display the distribution of shared gene families across selected species [55]. Gene ontology (GO) terms significantly enriched in *E. lecanii-corni* relative to other fungal species were identified using the GO term enrichment tool on the OrthoVenn2 platform. To evaluate orthologues underrepresented in *E. lecanii-corni*, orthogroups generated from OrthoFinder were assessed to identify orthogroups that lacked an *E. lecanii-corni* orthologue relative to either *E. dermatitidis* or the 17 other species included in this study [46,47]. GO term enrichment analysis was then carried out on *E. dermatitidis* genes present in these groups using FungiFun2 [56]. Secondary metabolite core biosynthesis genes, including polyketide synthases (PKSs), PKS-like enzymes, nonribosomal peptide synthetases (NRPSs), NRPS-like enzymes, and terpene cyclases (TCs) were predicated using antiSMASH version 5.0 [57].

## 3. Results

### 3.1. Phenotypic Analyses, Melanin Biosynthesis and Melanin Characterization in E. lecanii-corni

**Figure 1:**
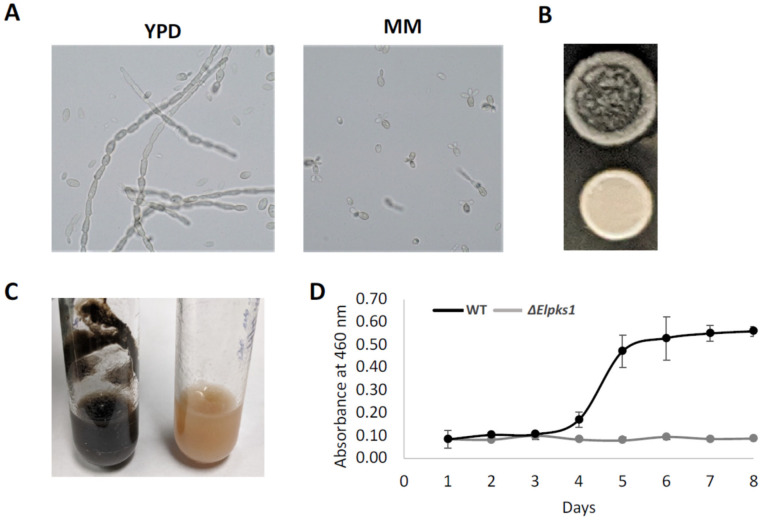
Phenotypes of *E. lecanii-cornii* melanin-producing WT and melanin-deficient mutant *Elpks1Δ*: (**A**) microscopic imaging dimorphic phenotypes of WT in YPD and MM; (**B**) colony morphology in agar plate; (**C**) biofilm formation in liquid YPD media; (**D**) melanization process in liquid YPD media.

Like many other black yeasts, the highly melanized *E. lecanii-corni* is dimorphic, exhibiting both yeast and hyphae forms in rich medium. Interestingly, however, the yeast form appears dominant when grown in minimal medium (Figure 1A). This indicates that the yeast-to-hyphae transition is regulated by environmental conditions. Melanin has been shown to regulate cell morphology [58], and therefore, in characterizing impact of melanin on the phenotypic properties of *E. lecanii-corni*, we generated a melanin deficient mutant by deleting *Elpks1*, a gene encoding the polyketide synthase responsible for melanin biosynthesis (Figure 1B). Melanin deficiency did not affect cell dimorphism (data not shown). However, in YPD agar, the macroscopic colony morphology of the wild type appeared strikingly different-more wrinkled and more invasive than that of the *Elpks1Δ* mutant. In liquid medium, moreover, the wild type strain tended to form biofilms along the surface, which did not occur in the non-melanized strain (Figure 1C). This result suggests that melanin enhanced cellular strength for penetration of substrates and promoted biofilm formation.

Given the broad range of functional properties and proposed applications of fungal melanin [25,26], we were interested in identifying a prolific melanin-producing fungus, and therefore, we next examined growth of *E. lecanii-corni* in a range of liquid media compositions to identify culture conditions that optimized melanin production yields. Media screened included yeast peptone dextrose (YPD), yeast extract glucose (YEG), yeast extract sucrose (YES), malt peptone glucose (MPG), malt extract (MB), Czapek (CZ), Czapek yeast (CY), and glucose minimal media optimized for yeast (GMM). *E. lecanii-corni* cells were inoculated into each medium and grown at 25 °C for 2 weeks, followed by extraction of crude melanin from culture supernatants by acidification and autoclaving. Of the conditions tested, melanin production was maximized when *E. lecanii-corni* cells were cultivated in MB liquid media, which exhibited a melanin production rate of approximately 3.2 g/L. High yields were also observed in MPG and YPD cultures, both of which produced approximately 2 g of melanin per 1 L culture media. Other conditions, including CY, YES, and YEG, were found to produce approximately 0.6 g/L, 0.4 g/L, and 0.1 g/L of melanin, respectively. Minimal melanin production was observed in both MM and CZA. These findings suggest that melanin production is varied in different growth conditions and optimized in malt- or peptone-based culture media. Moreover, measurement of OD_460_ intensities of the culture supernatants was used to monitor the rate of melanization of *E. lecanii-corni* in YPD broth at 25 °C over the course of eight days. Melanization exponentially increased between the fourth and fifth day and reached a plateau after the sixth day (Figure 1D).

**Figure 2:**
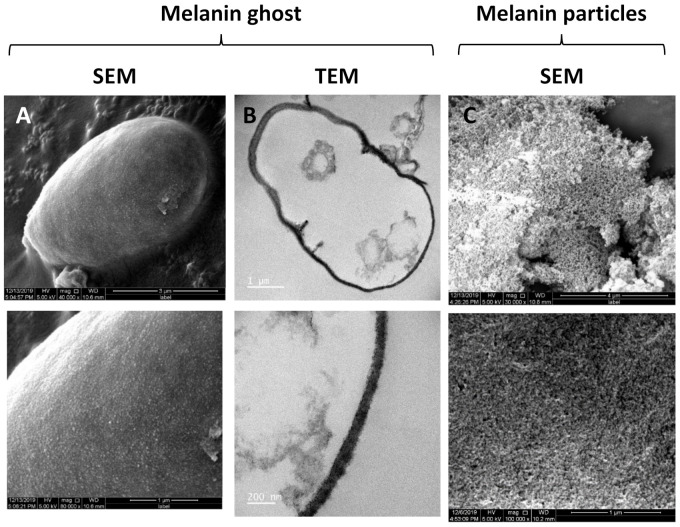
(**A**) FESEM and (**B**) TEM imaging melanin ghosts and (**C**) SEM imaging melanin particles.

Melanin synthesized from fungi is typically assembled within the cell wall and plays a significant role in maintaining cell wall integrity [59]. This means that melanin structures associated with the fungal cell walls, so-called “melanin ghosts”, can be generated, usually by treating melanized cells with 4 M guanidinium isothiocyanate followed by 6 M HCl at 100 °C. This protocol was applied to treat *E. lecanii-corni* wild type and *Elpks1Δ* mutant cells. The albino cells were completely solubilized but the wild type cells remained as black particulate materials. SEM images showed typical yeast morphologies with defined nano-sized melanin granules on the surface (Figure 2A). TEM revealed the hollow ghost structures and the melanin layer from the cell walls (Figure 2B), the density of which was substantially higher than that observed in melanin ghosts of *E. dermatitidis* [60], confirming that *E. lecanii-corni* synthesized significantly more melanin than *E. dermatitidis*. Additionally, the melanin particles precipitated from the black supernatant of cell culture with acid showed porous structures by SEM (Figure 2C).

### 3.2. Effect of Melanin Production on Stress Resistance in E. lecanii-corni

**Figure 3:**
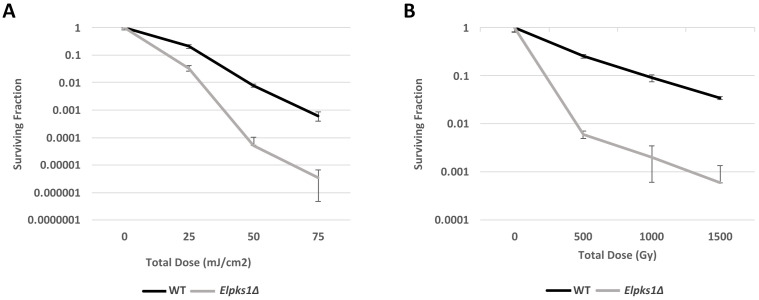
Survival rate of *E. lecanii-corni* WT and *Elpks1Δ* cells following exposure varying total doses of (**A**) UV-C radiation with dose rate of 0.273 mJ/cm^2^, and (**B**) γ-radiation with a dose rate of 36 Gy/min.

Given the well-established UV-protective properties of melanin in fungi [61,62,63,64], as well as some evidence that melanin may also confer protection to ionizing radiation, we next characterized the impact of melanin on resistance to UV- and γ-radiation in *E. lecanii-corni*. The melanin-producing wild type strain and melanin-deficient *Elpks1Δ* mutant strain were exposed to varying doses of UV-C and γ-radiation, ranging from 5–75 mJ/cm^2^ and 500–1500 Gy, respectively. This experiment yielded two key findings. First, we established that *E. lecanii-corni* has a D_10_ (the dose after which only 10% of cells survive) of approximately 1000 Gy (Figure 3A). While this is within the range of reported D_10_ values for other fungal species (500–2500 Gy) [65], it is still considerably lower than the closely related *Exophiala dermatitidis* (D_10_ = ~3000 Gy) [6]. Second, we found that deletion of the melanin biosynthesis gene leads to increased susceptibility to both UV-C and γ-radiation in *E. lecanii-corni* (Figure 3). Notably, melanin-deficiency resulted in an approximately 40-fold reduction in resistance to γ-radiation at doses of 500 and 1000 Gy, and this effect appears become more pronounced as dose increases, with 1500 Gy resulting in a 57-fold reduction in viability in the non-melanized strain (Figure 3B). These findings suggest that in this particular organism, melanin confers protection against both non-ionizing and ionizing forms of radiation.

**Figure 4:**
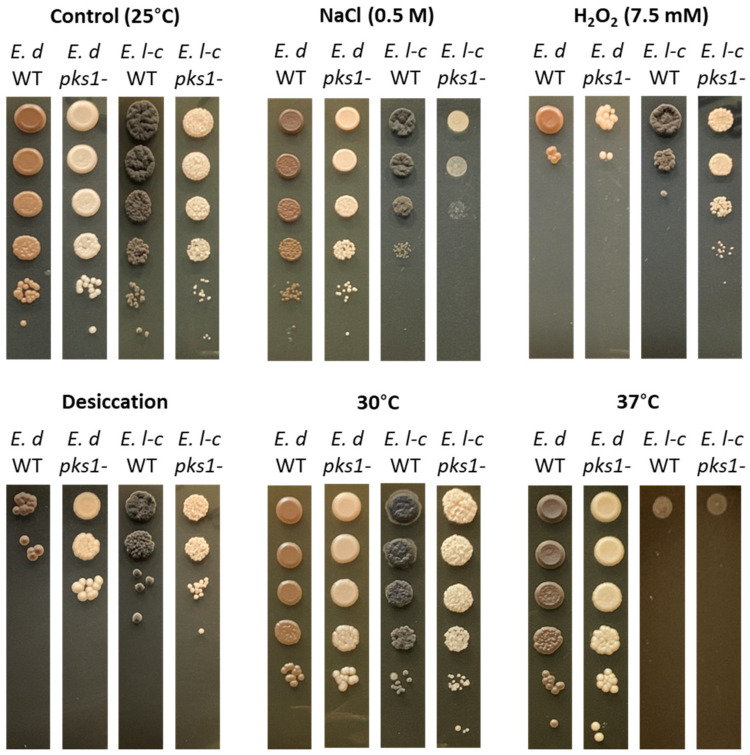
Relative survival of *E. dermatitidis* WT and *pks1Δ* cells and *E. lecanii-corni* WT and *Elpks1Δ* cells following exposure to osmotic (NaCl), oxidative (H_2_O_2_), desiccation, or temperature (30 °C and 37 °C) stress. Each stress assay was performed at least two times with two different biological replicates. Control refers to untreated sample.

We considered that melanin could affect several aspects of the fungal stress response, and so next we investigated the role of melanin in resistance of *E. lecanii-corni* to other forms of stress. In this case, we also performed a comparison with *E. dermatitidis*, as this latter organism has been shown to not require melanin for many of its stress responses [6]. This was accomplished by exposing the wild type and *pks1Δ* mutant strains of *E. lecanii-corni* and *E. dermatitidis* to osmotic (NaCl), oxidative (H_2_O_2_), desiccation, and temperature stress (30 °C and 37 °C) (Figure 4). The results revealed that *E. lecanii-corni* is substantially more susceptible to osmotic stress than *E. dermatitidis*, and that melanin contributes to osmoresistance in *E. lecanii-corni*. In contrast, *E. lecanii-corni* was found to be more resistant to oxidative stress than *E. dermatitidis*, with the effect being more pronounced in the melanin-deficient strain. *E. lecanii-corni* was also observed to be more resistant to desiccation relative to *E. dermatitidis*, and while melanin deficiency improved desiccation tolerance in *E. dermatitidis*, no such effect was observed with *E. lecanii-corni* (Figure 4). Lastly, while viability of both species is unaltered at 25 °C and 30 °C, growth at 37 °C leads to complete loss of viability in both the *E. lecanii-corni* wild type and non-melanized strains, despite this temperature being well-tolerated by *E. dermatitidis*. Notably, both desiccation and higher temperature enhanced melanin biosynthesis in *E. dermatitidis*.

### 3.3. Comparison of the E. lecanii-corni and E. dermatitidis Genomes

Having established key differences in the radiation- and stress-resistance phenotypes of the somewhat closely related black yeasts *E. lecanii-corni* and *E. dermatitidis*, we next aimed to investigate the genetics underlying these differences. The characteristics of each genome, as well as those of *E. mesophila* [38], the closest relative of *E. lecanii-corni*, are presented in Table 1. We recently reported the sequencing and assembly of the *E. lecanii-corni* genome [33], which revealed a 34.4 Mb genome containing 11,005 protein-coding sequences and 64 tRNAs. This is substantially larger than the genomes of *E. mesophila* (30.4 Mb, ~10,400 proteins) [38] and *E. dermatitidis* (26.4 Mb, ~9600 proteins). Similar to *E. dermatitidis*, and most other fungi, however, only a small fraction of the *E. lecanii-corni* genome consisted of repeat sequences, with a total of 660 elements comprising 2.7% of the genome (Table 2). Approximately one-third of these sequences were identified as retroelements, which consisted of both Ty1/Copia (63%) and Gypsy/DIRS1 (37%) types. DNA transposons represent 9.0% of the identified repeat sequences, with an approximate 2-to-1 ratio of PiggyBac to Tcl-IS630-Pogo types. Alternatively, while the genome of *E. dermatitidis* is comprised of a roughly similar proportion of repeat sequences, nearly half of them are retroelements, most of which are Gypsy/DIRS1 type, and just 3.3% of repeat sequences were identified as DNA transposons. Notably, transposable elements have been reported to drive adaptive evolution through genome restructuring in eukaryotic organisms [66], and pathogenic and animal-related fungi generally feature a higher percentage of transposable elements relative to fungi with different lifestyles [67].

### 3.4. Phylogenetic Characterization of E. lecanii-corni

**Figure 5:**
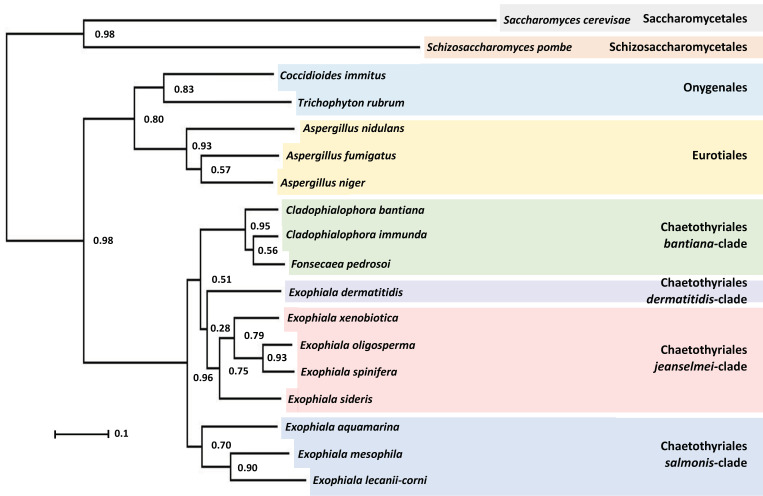
Rooted species tree inferred using OrthoFinder displaying the relative placement of *E. lecanii-corni* among the various clades of Chaetothyriales and other well-characterized ascomycetous species. Numbers at internal nodes refer to bootstrap support values.

To obtain a phylogenetic framework for the comparative genomic analyses, we assessed the phylogenetic relationship of *E. lecanii-corni* relative to other Chaetothyriales, including those within the *bantiana*-clade (*Cladophialophora bantiana*, *Cladophialophora immunda*, *Fonsecaea pedrosoi*), the *dermatitidis*-clade (*E. dermatitidis*), the *jeanselmei*-clade (*Exophiala xenobiotica*, *Exophiala oligoosperma*, *Exophiala spinifera*, *Exophiala sideris*), and the *salmonis*-clade (*Exophiala aquamarina*, *Exophiala mesophila*) [5]. For comparative purposes, we also included three well-characterized Eurotiales (*Aspergillus nidulans*, *Aspergillus fumigatus*, *Aspergillus niger*), two Onygenales (*Coccidioides immitis*, *Trichophyton rubrum),* and two other well-studied ascomycete models (*Saccharomyces cerevisiae*, *Schizosaccharomyces pombe*). For all the species listed above, OrthoFinder was used to identify a total of 13,838 orthogroups that represented approximately 94% of genes in the genomes (Supplemental Table S1), and from that data infer a rooted species tree (Figure 5) [46,47]. This revealed that *E. lecanii-corni* is situated in the *salmonis*-clade of the order Chaetothyriales, which is generally comprised of mesophilic waterborne *Exophiala* species [68]. Notably, while fungal species within this clade commonly cause infection in aquatic animals, such as fish, turtles, crabs, and frogs, they rarely infect humans, as their maximal growth temperature is approximately 33 °C [68]. Within this clade, *E. lecanii-corni* displays the closest phylogenetic relationship to *E. mesophila*, a black yeast with significant bioremediation potential due to its ability to digest alkylbenzene compounds [69].

### 3.5. Comparative Analysis of Predicted Functional Content

**Figure 6:**
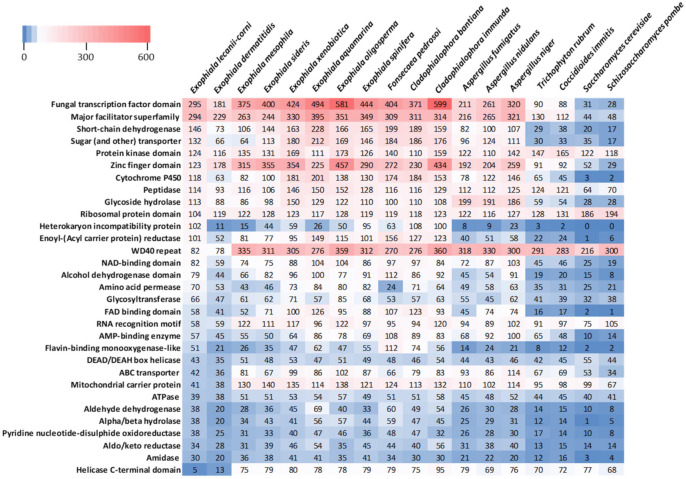
Pfam database protein domain count, as predicted using the PfamScan Perl script, in the *E. lecanii-corni* relative to other fungi included in this analysis.

Next, we examined variability in the predicted functional proteomic content of the *E. lecanii-corni* genome relative to the range of species included in the phylogenetic tree. To accomplish this, functional Pfam database protein families were identified for each of the species included in the analysis, and the relative occurrence of each protein domain or family was assessed (Figure 6). In the *E. lecanii-corni* genome, the most prevalent Pfam domains are for the categories of fungal transcription factor (TF) domain and major facilitator superfamily. However, while the genome of *E. lecanii-corni* harbors approximately 63% more TF domains than *E. dermatitidis*, it still generally has less TFs and zinc finger domains compared to other species within the Chaetothyriales order.

Relative to *E. dermatitidis*, *E. lecanii-corni* also possesses approximately twice as many short-chain dehydrogenases, sugar transporters, and cytochrome P450 domains, and most notably, encodes a relatively high number of heterokaryon incompatibility proteins compared to nearly all of the other species that were evaluated. On the other hand, the data also revealed that the genomes of *E. lecanii-corni* and *E. dermatitidis* both harbor substantially fewer WD40 repeats, RNA recognition motifs, mitochondrial carrier proteins, and helicases, and that *E. lecanii-corni* has an average of 20% fewer ribosomal protein domains compared to the other species included in the study (Figure 6).

**Figure 7:**
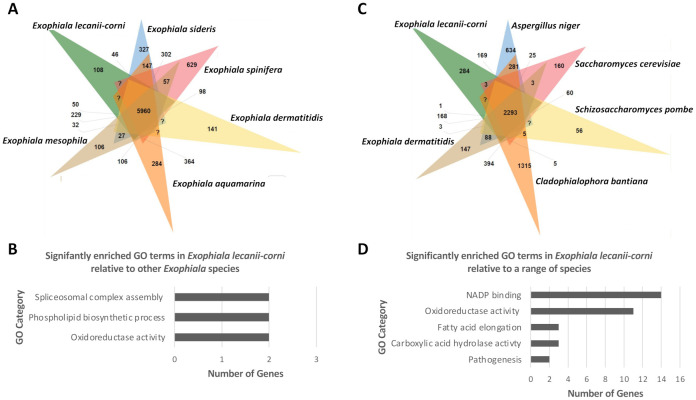
Venn diagrams displaying the distribution of shared orthologous gene clusters among (**A**) *E. lecanii-corni* and other *Exophiala* species, and (**C**) *E. lecanii-corni*, *E. dermatitidis*, *C. bantiana*, *S. cerevisiae*, *S. pombe*, and *A. niger*, as predicted using OrthoVenn. Significantly enriched GO categories associated with (**B**) the 108 gene cluster families that are specific to *E. lecanii corni* relative to *E. dermatitidis*, *E. sideris*, *E. spinifera*, *E. aquamarina*, and *E. mesophila*, and (**D**) the 284 gene cluster families that are specific to *E. lecanii corni* relative to *E. dermatitidis*, *C. bantiana*, *S. cerevisiae*, *S. pombe*, and *A. niger* (*p* < 0.05).

**Figure 8:**
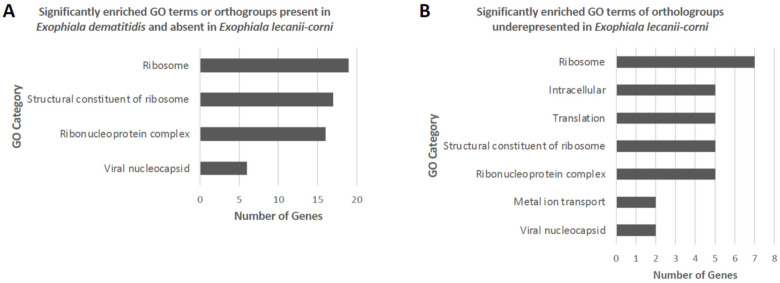
Significantly enriched (*p* < 0.05) GO categories of orthologues identified using OrthoFinder that are absent in *E. lecanii-corni* relative to (**A**) all species including in this analysis, and (**B**) *E. dermatitidis*.

To further investigate differences in the relative functional content of *E. lecanii-corni*, OrthoVenn2 was first used to comparatively evaluate orthologous gene clusters across species within the *Exophiala* genus (Figure 7A) [55]. The results indicated that relative to *E. dermatitidis*, *E. sideris*, *E. spinifera*, *E. aquamarina*, and *E. mesophila*, the genome of *E. lecanii-corni* harbors 5960 overlapping and 108 unique orthologous gene clusters. Gene Ontology (GO) enrichment analysis further revealed that among the 108 *E. lecanii-corni*-specific clusters, the GO categories of spliceosomal complex activity (two genes), phospholipid biosynthetic processes (two genes), and oxidoreductase activity (two genes) were significantly overrepresented (Figure 7B). When compared to a range of other fungal species, including *A. niger*, *S. cerevisiae*, *S. pombe*, *C. bantiana*, and *E. dermatitidis*, *E. lecanii-corni* was found to possess 284 unique orthologous gene clusters (Figure 7C) that feature significant overrepresentation of genes involved in NADP binding (14 genes) and oxidoreductase activity (11 genes) (Figure 7D). Furthermore, GO enrichment analysis conducted on orthogroups (Supplemental Table S1) (predicted by OrthoFinder [47]) that are absent in *E. lecanii-corni* but present in either *E. dermatitidis* (Figure 8A) or in all the species included in the previous analyses (Figure 8B) resulted in ribosomal constituents being significantly enriched, indicating that ribosomal functional activities are significantly underrepresented in *E. lecanii-corni* relative to a range of other ascomycetous fungal species.

### 3.6. Toluene Degradation Pathway Comparison

Given the potential for *E. lecanii-corni* to be utilized as a bioremediation agent for the degradation of volatile organic compounds [8], we next examined its genome for genes involved in toluene degradation [70]. The putative genes involved in the fungal toluene degradation pathway were previously identified in the toluene-degrading black yeast *C. immunda* based on sequence homology and regulation in response to toluene exposure [71]. Using pBLAST [72], we identified *E. lecanii-corni* homologs for all of the putative *C. immunda* toluene degradation pathway genes, displayed in Table 3. While many of the genes involved in the fungal toluene degradation pathway are clustered in *C. immunda*, this trend was substantially less pronounced in *E. lecanii-corni*. In *C. immunda*, four of the putative genes encoding benzylalcohol dehydrogenase (BADH) and (benzaldehyde dehydrogenase) BZDH enzymes are grouped in two separate clusters and seven of the genes involved in protocatechuate degradation are grouped in two additional clusters [71]. In contrast, in the *E. lecanii-corni* genome, only two genes, EXLC_001487T0 (carboxy-muconolactone decarboxylase (CMD)) and EXLC_001488T0 (β-carboxy-muconate lactonizing enzyme (CMLE)), were observed to be clustered.

### 3.7. Melanin Biosynthesis and Regulation Pathway Comparison

We next examined the genome of *E. lecanii-corni* for genes known to be involved in the production of melanin, as it is distinguished by its high capacity for melanin production. Most fungi produce either 1,8-dihydroxynaphthalene (DHN)-melanin or 3,4-dihydroxyphenylalanine (DOPA)-melanin, with ascomycetous fungi generally utilizing the DHN pathway and basidiomycetous fungi commonly using the DOPA pathway [73]. Biosynthesis of DHN-melanin initiates with the catalyzed formation of 1,3,6,8-tetrahydroxynaphthalene (1,3,6,8-THN) from malonyl-CoA and acetyl-CoA by a polyketide synthase (PKS). Next, a series of enzymatic reactions catalyzed by scytalone dehydratase, 1,3,6,8-tetrahydroxynaphthalene reductase, alpha/beta hydrolases, multicopper oxidases, ferroxidase led to the formation of DHN. Finally, polymerization of DHN, which may be catalyzed by a laccase, generates DHN-melanin [74,75]. We identified *E. lecanii corni* homologs for all the enzymes involved in melanin biosynthesis, which are displayed in Table 4 alongside *E. dermatitidis* homologs [27]. The genome of *E. lecanii-corni* harbors two homologs of the *E. dermatitidis* melanin-producing PKS, along with a homolog of alpha/beta hydrolase Ayg1, both of which are required for DHN-melanin biosynthesis in *E. dermatitidis* [76,77]. However, it was previously reported that only one of these *E. lecanii-corni* PKSs, EXLC_009455T0 (the gene that results in a non-melanized phenotype when deleted), is involved in melanin production, while the function of the other PKS remains unknown [10]. Similar to the *E. dermatitidis* genome, *E. lecanii-corni* possesses a single homolog of scytalone dehydratase Arp1 and alpha/beta hydrolase HMPREF1120_02312, along with two homologs laccase Abr2 and L-ascorbate oxidase. In contrast, we identified two homologs of 1,3,6,8-tetrahydroxynaphthalene reductase Arp2, relative to the single homolog present in *E. dermatitidis*. We also noted that ferrooxidoreductase is underrepresented in *E. lecanii-corni* relative to *E. dermatitidis*, which harbor three homologs. Lastly, similar to *E. dermatitidis*, the genes involved in melanin biosynthesis in *E. lecanii-corni* are not clustered together in the genome as they are in many other fungal species [78].

Ascomycetous fungi typically produce DHN-melanin, but several members have been reported to produce DOPA-melanin, including *Aspergillus nidulans*, *Hypoxylon archeri*, *Penicillium marneffei*, and *Sporothrix schenckii* [79,80,81,82]. Other fungi, such as the edible mushroom *Auricularia auricula*, have been reported to produce a mixture of DHN- and DOPA-melanin [83]. Biosynthesis of DOPA-melanin in fungi resembles human melanogenesis, initiating with the catalyzed oxidation of either DOPA to dopaquinone by laccase, or tyrosine to DOPA to dopaquinone by tyrosinase [58,74]. Although DHN-melanin is the form of melanin constitutively produced by *E. lecanii-corni* [10], its genome also contains genes required for the production of DOPA-melanin (Table 4). We identified five tyrosinase homologs and seven multicopper oxidases in the *E. lecanii-corni* genome, which is comparable to the numbers present in *E. dermatitidis*, as well as other melanized fungal species, such as *A. niger* [27]. 

Finally, the genes involved in the production of a third type of melanin, pyomelanin, are also conserved in *E. lecanii-corni* (Table 4). Pyomelanin production involves polymerization of homogentisic acid of the tyrosine degradation pathway, and is conserved among other ascomycetous fungi, including *E. dermatitidis*, *A. fumigatus*, and *Penicillium chrysogenum* [84,85]. More specifically, its biosynthesis involves the catalyzed formation of 4-hydroxyphenyl pyruvate from tyrosine-by-tyrosine aminotransferase Tat, followed by conversion to homogentisate by 4-hydroxyphenylpyruvate dioxygenase HppD, and subsequent oxidation and polymerization to form pyomelanin. Alternatively, homogentisate can be degraded by homogentisate dioxygenase HmgA, maleylacetoacetate isomerase MaiA, and fumarylacetoacetate hydrolase FahA to yield acetoacetate and fumarate [84]. Our analysis revealed that the genome of *E. lecanii-corni* harbors single homologs of Tat, HppD, and MaiA, along with six and three homologs of HmgA and FahA, respectively, which is in contrast to the single homolog present in *E. dermatitidis* (Table 4).

### 3.8. Potential for Secondary Metabolite Production

Many fungal species are potent producers of secondary metabolites (SMs), which are small bioactive molecules that confer selective advantage while not being directly required for survival [86]. These small molecules possess a broad range of bioactivities that have resulted in their widespread use in medicine and industry [87,88]. The genes involved in the biosynthesis of SMs are typically clustered together in the genome and include a core biosynthesis enzyme, such as a PKS or PKS-like enzyme, a nonribosomal peptide synthetase (NRPS) or NRPS-like enzyme, or a terpene cyclase (TC), along with various tailoring enzymes. To evaluate the relative potential for SM production in *E. lecanii-corni*, we identified core SM biosynthesis enzymes in all species included in this study using antiSMASH [57]. This resulted in the identification of five PKS or PKS-like enzymes, seven NRPS of NRPS-like enzymes, and three TCs, or a total of fifteen SM core enzymes in the genome of *E. lecanii-corni* (Table 5). While this number is significantly higher than the number of SMs produced by some yeast species, such as *S. cerevisiae* and *S. pombe*, and comparable to other *Exophiala* species, it is considerably less than other ascomycetous fungi, including many within the *Aspergillus* genus [89] (Table 5). Of the fifteen SM core synthase enzymes identified in *E. lecanii corni*, only four possessed a high percentage of genes that have significant BLAST hits to genes present in characterized clusters with known SM products [57]. These clusters are involved in the production of nonribosomal peptides dimethylcoprogen (100% cluster similarity) and acetylaranotin (80% cluster similarity), the terpene clavaric acid (100% cluster similarity), and the polyketide 1,3,6,8-tetrahydroxynaphthalene involved in DHN-melanin production, indicating that these SM biosynthesis pathways are conserved in *E. lecanii-corni*.

## 4. Discussion

In this study, we conducted a phenotypic and comparative genomic analysis of the melanin-producing yeast *E. lecanii-corni* to acquire insight into the biological basis for differences in stress tolerance and pathogenicity among black yeast. Toward this goal, we have successfully identified key differences in stress tolerance of *E. lecanii-corni* relative to the well-characterized *E. dermatitidis*, as well as the impact of melanin on these phenotypes. Further, we uncovered variability in genetic functional content that may explain some observed differences in extremophilic characteristics. These studies have contributed to our understanding of the biology of black yeast and provide a framework for targeted functional studies that will advance our understanding of this unique organism.

Our investigation revealed that the presence of melanin significantly increased the resistance of *E. lecanii-corni* to acute doses of UV-C and γ-radiation. This finding was expected for UV-C radiation, as the capacity of melanin to absorb radiation at a broad range of wavelengths within the UV and visible electromagnetic spectrum is well understood [21], and several studies have consistently found that melanin confers protection against UV radiation in fungi [61,62,63,64]. On the other hand, the involvement of melanin in resistance to ionizing radiation is less clear, which makes our finding, that melanin confers protection against γ-radiation as well, particularly notable. While some studies have observed a protective effect of melanin against ionizing radiation, those findings have been mixed, with any impact often being marginal [90,91] or entirely absent [6,64,92]. Other investigations have revealed a more complicated role for melanin in the fungal response to ionizing radiation. For example, one study found that in *E. dermatitidis*, the presence of melanin altered the regulation of specific types of genes, including those encoding transporters and ribosomal biosynthesis enzymes, in response to low-dose ionizing radiation [93]. In contrast, a different study that examined the response of *Cryptococcus neoformans* to ionizing radiation found that melanin did not have a substantive impact on gene expression patterns [92]. Together, these findings suggest that the impact of melanin on fungal radiation resistance is likely species-specific and can depend on a range of other factors, including total dose, dose rate, the physiological conditions of the fungi prior to exposure, and the distinct physico-chemical properties of the melanin variant being produced. Further, it is unclear the extent to which the presence or absence of melanin alters other physiological parameters that can impact the ability of fungi to survive and recover from ionizing radiation exposure. Since the presence of melanin on gene expression appears to vary substantively between organisms [6,92], studies that aim to quantify the true ionizing radiation protective properties of melanin should consider conducting such experiments outside the constraints of biological systems, which can convolute the findings. Still, the effect is substantial for *E. lecanii-corni* melanin and should be explored further.

Similarly, our study also revealed that the impact of melanin on tolerance to other forms of stress can also vary drastically, even among closely related species. For example, we observed that while melanin deficiency does not alter oxidative stress resistance in *E. dermatitidis*, *E. lecanii-corni* strains lacking melanin production are substantially more resistant to oxidative stress due to H_2_O_2_ than their melanized counterparts. Although the antioxidative properties of melanin make this finding somewhat counterintuitive, it may be explained by the fact that melanin production represents a significant metabolic cost to the cell. It is therefore possible that the saved energy expenditures from lack of melanin production can be redirected to activate biological pathways that are critical for ionizing radiation resistance, such as those involved in oxidative stress protection and DNA repair. This phenomenon may be more pronounced in *E. lecanii-corni* than in *E. dermatitidis* because *E. lecanii-corni* produces a relatively larger quantity of melanin, and is therefore, burdened by a higher metabolic load. In contrast, melanin was found to enhance resistance to osmotic stress in *E. lecanii-corni*, which is in agreement with other studies that have examined the impact of cell wall DHN-melanin on osmotic stress resistance in other ascomycetous fungi [11]. Rigid structure and high-density melanin nanoparticles enriched in cell wall as demonstrated in the melanin ghosts (Figure 2) may contribute to resistance of melanized cells to osmotic stress. Interestingly, no such impact was observed in *E. dermatitidis*, which also may be due to its decreased abundance of cell wall melanin relative to *E. lecanii-corni*.

We were particularly interested in understanding the biological basis underlying the reduced human pathogenicity of *E. lecanii-corni*, as this characteristic makes this organism attractive for functional laboratory studies or production host capabilities in industry. In order for a fungus to successfully invade the human body, it must be capable of thriving in a range of extreme environments, including the high temperatures associated with vertebrate endothermy [94], low pH and water if penetration occurs through the skin [95], and reactive oxygen species produced by phagocytes [96]. We observed that *E. lecanii-corni* CBS 102400 is entirely unable to grow at the physiological temperature of 37 °C, which distinguishes it from other *Exophiala* species, most of which grow at 37–40 °C [68]. This is considered to be a key virulence factor within the order Chaetothyriales [1], and helps to explain why this strain of *E. lecanii-corni* was categorized as BSL1. Notably, other *Exophiala* species that lack thermotolerance have been isolated, and many of these isolates are capable of causing disease in cold-blooded animals [68].

This investigation revealed distinct differences in functional genomic content that may play a role in stress tolerance variation. In particular, our most statistically significant observation was that the genome of *E. lecanii-corni* exhibited an approximate 20% reduction in genes encoding ribosomal proteins relative to the other ascomycetous species included in this study. Ribosomal proteins are the structural constituents of ribosomes, which are the cellular machinery responsible for the translation of mRNA to protein synthesis. Ribosomal gene expression is closely correlated with fungal growth and proliferation, as species harboring higher ribosome content typically exhibit increased growth rates [97,98]. Accordingly, we observed that *E. lecanii-corni* exhibits a slower growth rate than other black yeast, such as *E. dermatitidis* (data not shown). Interestingly, several studies have indicated that some ribosomal proteins have additional functions that are independent of their primary role of protein biosynthesis, also referred to as “moonlighting”, that can include regulation of cell proliferation, induction of apoptosis, cell signaling, and DNA repair [99,100]. More recently, the moonlighting functions of ribosomal proteins in fungi have been extended to include the conferral of tolerance to various forms of abiotic stress, including those imposed by salt, drought, and heavy metals [101]. In a similar vein, we previously observed that increased levels of ribosomal proteins were associated with the acquisition of ionizing radiation resistance in the black yeast *E. dermatitidis* [29]. This may be due to the fact that some proteins required for cellular recovery from radiation exposure are not synthesized until after exposure [6], and therefore, a strain with higher ribosomal content will have an increased capacity to maintain synthesis of key proteins required for survival following irradiation. On the other hand, this observation may also be another example of a novel ribosomal protein moonlighting function that involved the conferral of resistance to ionizing radiation. Either way, there is sufficient evidence to suggest that the significantly reduced ribosomal protein content in the genome of *E. lecanii-corni* may contribute to differences in its stress tolerance phenotype, including increased susceptibility to osmotic stress and ionizing radiation, which will be a topic of investigation in future studies.

Given the reduced pathogenicity of *E. lecanii-corni* CBS 102400 relative to other black yeast, a goal of this study was to provide a genetic framework that will advance the utilization of *E. lecanii-corni* for biotechnological applications. Toward this goal, we identified *E. lecanii-corni* homologues for the 13 putative genes belonging to the toluene degradation pathway in fungi. Notably, *E. lecanii-corni* has demonstrated bioremediation potential, as it is capable of using toluene as its sole carbon source and can eliminate toluene at a rate that is two to seven times greater than those reported for bacterial biofiltration systems [8]. This is a significant finding, as the capacity to degrade toluene is quite rare in fungi and is often strain-specific rather than species-specific [102,103]. Future efforts that aim to further develop *E. lecanii-corni* for bioremediation applications, including the degradation of volatile organic compounds, should consider targeting the putative toluene degradation pathway genes identified in this study.

Finally, this study illuminated the suitability for *E. lecanii-corni* to be utilized as a melanin production host for biotechnological applications. As expected, we identified homologs for all the genes involved in the biosynthesis of DHN-melanin, and in addition, we found that *E. lecanii-corni* also harbors the genes required for production of DOPA-melanin and pyomelanin. Similar to *E. dermatitidis*, the genes involved in DHN-melanin production are not clustered within the *E. lecanii-corni* genome as they are in other fungal species, reiterating previous findings that the coordinated regulation required for abundant melanin production can be achieved without gene clustering. DHN-melanin production was maximized when *E. lecanii-corni* was cultured in malt-based media, with a production yield of approximately 3.2 g/L, which represents a remarkable production rate. A number of native and recombinant microorganisms have been reported to produce melanin at relatively high yields, but they all produce eumelanin (DOPA-melanin) and require supplying tyrosine as the precursor in growth media [104]. There still remains significant potential for enhancing melanin production efficiency in *E. lecanii-corni*, either through optimization of fermentation conditions or engineering of biosynthetic pathways [105]. Future endeavors that require large quantities of DHN-melanin for characterization studies or other biotechnological applications should consider the utilization and development of *E. lecanii-corni* for such purposes.

## 5. Conclusions

Taken together, this study highlights the effectiveness of combining phenotypic characterization with comparative genomic analysis to identify candidate genetic signatures that may contribute to a particular stress tolerance phenotype in fungi. Using this approach, we have uncovered the significant underrepresentation of ribosomal genes within the *E. lecanii-corni* genome, providing insight into the biological basis for its distinct stress tolerance profile, as well as a starting point for future studies investigating how ribosomal content alters the characteristics of a particular organism. Further, we have provided a detailed account of the functional genomic content within the genome of *E. lecanii-corni*, paving the way for targeted functional studies and its use as a production host for melanin or other useful biomolecules.

## Figures and Tables

**Table 1:** General genome characteristics and assembly features of *E. lecanii-corni* CSB 102400 compared to *E. mesophila* CCFEE 6314 [38] and *E. dermatitidis* NIH/UT8656.

Genome Feature	*Exophiala lecanii-corni*	*Exophiala mesophila*	*Exophiala dermatitidis*
	CBS 102400	CCFEE 6314 [38]	NIH/UT8656
Size (Mb)	34.42	30.43	26.38
GC content (%)	48.94	50	51.47
Scaffolds	13	207	11
N50 (Mb)	2.95	0.43	3.62
tRNA (#)	64	38	73
Protein-coding genes (#)	11,005	10,355	9562

**Table 2:** Distribution of classified repeat elements within the genomes of *E. lecanii-corni* and *E. dermatitidis*.

		*Exophiala lecanii-corni*			*Exophiala dermatitidis*		
		Number of Elements	% of Genome Sequences	% of Repeat Sequences	Number of Elements	% of Genome Sequences	% of Repeat Sequences
Retro- elements	LINEs	0	0%	0%	14	0.21%	5.8%
	Ty1/Copia (LTR)	76	0.78%	20.8%	53	0.30%	8.4%
	Gypsy/DIRS1 (LTR)	59	0.45%	12.0%	128	1.09%	30.4%
	Total	135	1.23%	32.8%	195	1.60%	44.7%
DNA transposons	Tcl-IS630-Pogo	103	0.12%	3.2%	30	0.12%	3.3%
	PiggyBac	39	0.22%	5.90%	0	0.00%	0.00%
	Total	142	0.34%	9.0%	30	0.12%	3.3%
Unclassified		383	1.13%	30.0%	811	0.95%	26.7%
Total		660	2.70%	71.70%	1036	2.67%	74.70%

**Table 3:** Toluene degradation pathway genes present in the genome of *E. lecanii-corni* relative to *C. immunda*.

Gene Annotation	*E. lecanii-corni*	*C. immunda* CBS 110551	*C. immunda* CBS 834.96	BLAST Score (Bits)	E-Value
Cytochrome P450	EXLC_010870T0	CLAIMM_07379	XP_016251043.1	723	0.0
Cytochrome P450	EXLC_003659T0	CLAIMM_09044	XP_016247671.1	565	0.0
Cytochrome P450	EXLC_001047T0	CLAIMM_11603		519	0.0
Cytochrome P450	EXLC_007222T0	CLAIMM_10796	XP_016250278.1	668	0.0
Benzylalcohol dehydrogenase (BADH)	EXLC_009515T0	CLAIMM_14566		317	1 × 10^−70^
Benzylalcohol dehydrogenase (BADH)	EXLC_000716T0	CLAIMM_12646		290	4 × 10^−96^
Benzylalcohol dehydrogenase (BADH)	EXLC_010898T0	CLAIMM_02069		350	2 × 10^−119^
Benzaldehyde dehydrogenase (BZDH)	EXLC_003682T0	CLAIMM_14573	XP_016255229.1	536	0.0
Benzaldehyde dehydrogenase (BZDH)	EXLC_007394T0	CLAIMM_12645		410	4 × 10^−140^
Benzaldehyde dehydrogenase (BZDH)	EXLC_003083T0	CLAIMM_07604		310	4 × 10^−101^
Benzoate hydroxylase (BH)	EXLC_000444T0	CLAIMM_00094	XP_016243286.1	936	0.0
p-hydroxybenzoate hydroxylase (PHBH)	EXLC_002510T0	CLAIMM_03385		1021	0.0
Decarboxylase (PCAD)	EXLC_008460T0	CLAIMM_02205		339	2 × 10^−111^
Dioxygenase (P34O)	EXLC_010602T0	CLAIMM_02325	XP_016246009.1	517	0.0
Catechol 1,2-dioxygenase (C12O)	EXLC_006633T0	CLAIMM_07423		234	1 × 10^−75^
Catechol 1,2-dioxygenase (C12O)	EXLC_005860T0	CLAIMM_03467	XP_016253603.1	336	1 × 10^−115^
β-carboxy-muconate lactonizing enzyme (CMLE)	EXLC_001488T0	CLAIMM_03466		285	6 × 10^−96^
β-carboxy-muconate lactonizing enzyme (CMLE)	EXLC_010962T0	CLAIMM_08374		112	1 × 10^−32^
Carboxy-muconolactone decarboxylase (CMD)	EXLC_001487T0	CLAIMM_03468	XP_016253602.1	203	2 × 10^−67^
Carboxy-muconolactone decarboxylase (CMD)	EXLC_004832T0	CLAIMM_07415	XP_016251077.1	73.9	6 × 10^−15^
Muconate lactonizing enzyme (MLE)	EXLC_006601T0	CLAIMM_14446	XP_016251156.1	449	6 × 10^−157^
β-ketoadipate-enol-actone hydrolase (ELH)	EXLC_008892T0	CLAIMM_05933		417	1 × 10^−149^
β-ketoadipate succinyl-CoA transferase (TR)	EXLC_006182T0	CLAIMM_07426		645	0.0
β-ketoadipyl-CoA thiolase (TH)	EXLC_000315T0	CLAIMM_07421		427	2 × 10^−148^

**Table 4:** Melanin biosynthesis pathway genes present in the genome of *E. lecanii-corni* compared to *E. dermatitidis*.

Gene	Annotation	*Exophiala lecanii-corni*	*Exophiala dermatitidis*
**DHN-melanin biosynthetic pathway**			
Pks1	Polyketide synthase	EXLC_004613T0, EXLC_009455T0	HMPREF1120_03173
Arp1	Scytalone dehydratase	EXLC_004054T0	HMPREF1120_07724
Arp2	1,3,6,8-tetrahydroxynaphthalene reductase	EXLC_001201T0, EXLC_006778T0	HMPREF1120_05939
Ayg1	Alpha/beta hydrolase	EXLC_008577T0	HMPREF1120_00377
	Alpha/beta hydrolase	EXLC_003727T0	HMPREF1120_02312
Abr2	Laccase	EXLC_004610T0, EXLC_010027T0	HMPREF1120_02828, HMPREF1120_05645
Abr1	Ferrooxidoreductase	EXLC_006706T0	HMPREF1120_00173, HMPREF1120_01590, HMPREF1120_04510
	L-ascorbate oxidase	EXLC_003729T0, EXLC_006667T0	HMPREF1120_03706, HMPREF1120_04536
**DOPA-melanin biosynthetic pathway**			
MelC2	Tyrosinase	EXLC_006713T0, EXLC_009460T0, EXLC_010702T0	HMPREF1120_03345, HMPREF1120_04514, HMPREF1120_05316
MelO	Tyrosinase	EXLC_000450T0, EXLC_009626T0	HMPREF1120_07692
MelO	Multicopper oxidase	EXLC_005401T0, EXLC_010629T0	HMPREF1120_05865
MelO	Multicopper oxidase	EXLC_001274T0	HMPREF1120_00199
MelO	Multicopper oxidase	EXLC_002340T0, EXLC_008951T0	HMPREF1120_08116
MelO	Multicopper oxidase	EXLC_009732T0, EXLC_009741T0	HMPREF1120_02754, HMPREF1120_04578, HMPREF1120_08564
**L-tyrosine degradation pathway**			
Tat	Tyrosine aminotransferase	EXLC_000526T0	HMPREF1120_02164
HppD	4-Hydroxyphenylpyruvate dioxygenase	EXLC_000706T0	HMPREF1120_05584
HmgA	Homogentisate dioxygenase	EXLC_000997T0, EXLC_002037T0, EXLC_006635T0, EXLC_007332T0, EXLC_008087T0, EXLC_010843T0	HMPREF1120_03827
FahA	Fumarylacetoacetate hydrolase	EXLC_000996T0, EXLC_002038T0, EXLC_007334T0	HMPREF1120_03825
MaiA	Maleylacetoacetate isomerase	EXLC_009316T0	HMPREF1120_03438

**Table 5:** Count of secondary metabolite core synthase genes in the genomes of *E. lecanii-corni* and other ascomycetous fungal species.

	PKS/PKS-Like	NRPS/NRPS-Like	TC	Total
***Aspergillus niger***	46	35	7	88
***Aspergillus nidulans***	33	25	2	60
***Coccidioides immitis***	10	11	4	25
***Exophiala aquamarina***	7	12	6	25
***Fonsecaea pedrosoi***	4	11	5	20
***Cladophialophora bantiana***	4	10	5	19
***Exophiala spinifera***	5	11	3	19
***Exophiala dermatitidis***	3	8	4	15
***Exophiala lecanii-corni***	5	7	3	15
***Exophiala mesophila***	2	7	4	13
***Schizosaccharomyces pombe***	0	3	1	4
***Saccharomyces cerevisiae***	0	1	1	2

## Data Availability

The raw genome sequencing reads for *E. lecanii-corni* CBS 102400 are available in NCBI SRA under Accession Number SRX5145602, as previously described [33].

## Supplementary Materials

The following are available online at https://www.mdpi.com/article/10.3390/jof7121078/s1, Supplemental Table S1. Orthologous gene clusters identified in this study, as predicted using OrthoFinder.
