# Supplementary material for: Phenotypic Characterization and Comparative Genomics of the Melanin-Producing Yeast Exophiala lecanii-corni Reveals a Distinct Stress Tolerance Profile and Reduced Ribosomal Genetic Content
Source: J Fungi (Basel). 2021 Dec 15;7(12):1078. doi: 10.3390/jof7121078 (PMC8709033; doi:10.3390/jof7121078)
Supplement: Supplementary file 1 [file jof-07-01078-s001.zip › jof-1453502-supplementary.pdf]

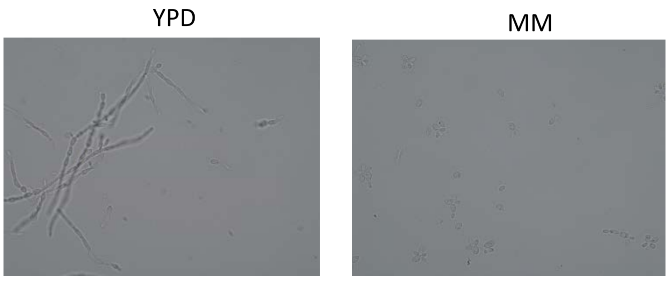

Supplemental Figure S1: microscopic imaging dimorphic phenotypes of melanin-deficient mutant *Elcpks1Δ* in YPD and MM
